# The built-in electric field across FeN/Fe_3_N interface for efficient electrochemical reduction of CO_2_ to CO

**DOI:** 10.1038/s41467-023-37360-9

**Published:** 2023-03-28

**Authors:** Jie Yin, Jing Jin, Zhouyang Yin, Liu Zhu, Xin Du, Yong Peng, Pinxian Xi, Chun-Hua Yan, Shouheng Sun

**Affiliations:** 1 State Key Laboratory of Applied Organic Chemistry, College of Chemistry and Chemical Engineering, Lanzhou University, Lanzhou, China; 2 Department of Chemistry, Brown University, Providence, RI USA; 3 Electron Microscopy Centre of Lanzhou University, Lanzhou University, Lanzhou, China; 4 College of Chemistry, Zhengzhou University, Zhengzhou, China; 5 State Key Laboratory of Rare Earth Materials Chemistry and Applications, Peking University, Beijing, China

**Keywords:** Electrocatalysis, Electrochemistry, Electrocatalysis

## Abstract

Nanostructured metal-nitrides have attracted tremendous interest as a new generation of catalysts for electroreduction of CO_2_, but these structures have limited activity and stability in the reduction condition. Herein, we report a method of fabricating FeN/Fe_3_N nanoparticles with FeN/Fe_3_N interface exposed on the NP surface for efficient electrochemical CO_2_ reduction reaction (CO_2_RR). The FeN/Fe_3_N interface is populated with Fe−N_4_ and Fe−N_2_ coordination sites respectively that show the desired catalysis synergy to enhance the reduction of CO_2_ to CO. The CO Faraday efficiency reaches 98% at −0.4 V vs. reversible hydrogen electrode, and the FE stays stable from −0.4 to −0.9 V during the 100 h electrolysis time period. This FeN/Fe_3_N synergy arises from electron transfer from Fe_3_N to FeN and the preferred CO_2_ adsorption and reduction to *COOH on FeN. Our study demonstrates a reliable interface control strategy to improve catalytic efficiency of the Fe–N structure for CO_2_RR.

## Abstract (web summary)

Understanding and controlling chemical environment of metal-N-catalysts is of great importance. In this work, the authors reveal FeN/Fe_3_N interface with Fe-N_4_ and Fe-N_2_ coordination sites for enhanced electrochemical CO_2_ reduction to CO.

## Introduction

Converting CO_2_ back to carbon (C)-based chemicals/fuels is a critical step en route to realizing green environment and energy sustainability^1–5^. CO_2_ can in principle be reduced to active forms of C-products, but the activated CO_2_ intermediates tend to undergo different reaction pathways, making it difficult to efficiently control the reaction selectivity^6–8^. Recently, the electrochemical CO_2_ reduction reaction (CO_2_RR) has been studied extensively due to its appealing potential to reduce CO_2_ with renewable electricity^9,10^. The center of these studies lies in the development of robust catalysts to lower the reduction overpotentials and to direct reaction pathways towards specific C-products. Among all possible products generated from the CO_2_RR, CO is an important precursor that is widely used for preparing other chemicals and fuels^11–13^. In the CO_2_RR process, CO is produced via two electron reduction of CO_2_^14,15^, and this reaction can be catalyzed by many different catalysts, including not only noble metals (Au, Ag, and Pd)^16^, but also non-noble metals (Cu, CuIn alloys)^17,18^, and metal−N_4_ (M−N_4_) complex structures^19–21^, as summarized in Supplementary Table 1. The M−N_4_ structures, especially Ni−N- and Fe−N-based structures, have attracted particular interest as a class of catalysts for CO_2_RR to CO due to increased electron conductivity with decreased band gap of M−N_4_ structures and intensive bonding interactions between *COOH intermediates and metal centers, thus lowering the overall reaction barriers and promoting CO generation. However, the Ni−N-complex catalysis is limited by slow kinetics of CO_2_ + H^+^ + e^−^ → *COOH and weak *H binding for necessary hydrogenation steps, while the Fe−N-complex catalysis is hindered by the strong *CO and *H binding to the Fe−N sites, which prevent CO from being released as a gas form, or from being further activated for the following hydrogenation steps^20,22–24^. Therefore, it is important to stabilize the chemical environment of metal-N-catalysts and to optimize their catalysis towards hydrogenation or desorption of CO at low overpotentials for successful CO_2_RR.

Here, we report a strategy to improve Fe−N catalysis efficiency for CO_2_RR. The catalyst is based on FeN/Fe_3_N heterostructure nanoparticles (NPs) in which Fe is coordinated by 4 N’s and 2 N’s respectively, forming a unique Fe−N_4_/Fe−N_2_ interface on the NP surface. This interface offers the desired synergetic effect on CO_2_ adsorption on FeN, and electron transfer from Fe_3_N to FeN, enhancing the CO_2_ reduction to CO. In the CO_2_-saturated 0.5 M KHCO_3_ solution, the catalyst shows a high CO Faradaic efficiency (FE) of 98% at −0.4 V (vs. RHE) without noticeable efficiency change in the 100 h electrolysis test between −0.4 V and −0.9 V. The catalysis produces CO with a high single-pass conversion rate up to 56%, which is unprecedented. Our study presents an interface design strategy for improving CO_2_RR catalysis efficiency.

## Results

**Fig. 1:**
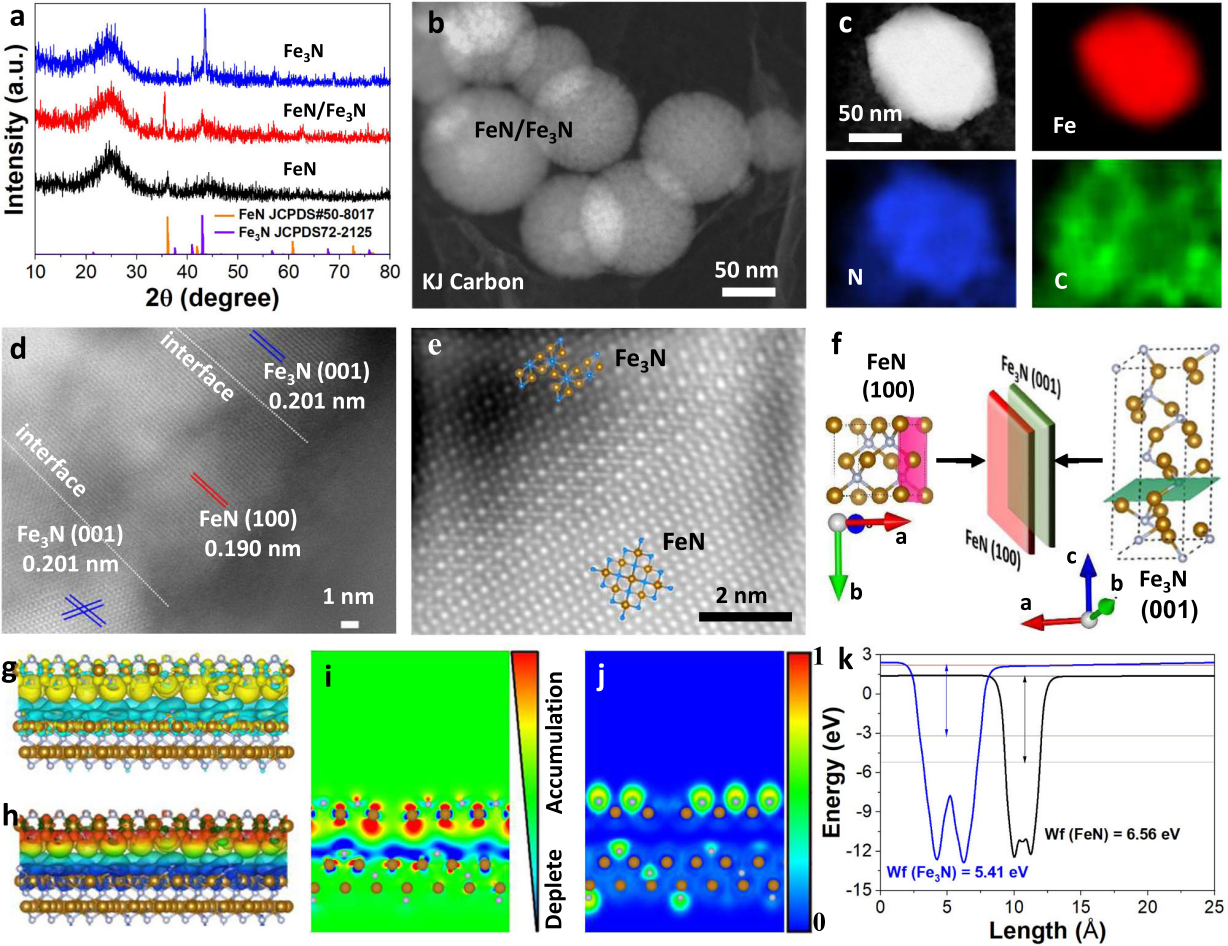
Fe-N NP characterization. **a** XRD patterns of FeN, FeN/Fe_3_N, and Fe_3_N NPs. **b** STEM image of FeN/Fe_3_N NPs. **c** Elemental mapping of Fe, N and C of a single FeN/Fe_3_N NP. **d** HRTEM image of FeN/Fe_3_N NPs showing the interface between FeN and Fe_3_N domains. **e** HRHAADF-STEM image of the FeN/Fe_3_N interface. **f** Schematic illustration of the FeN/Fe_3_N interface formed between FeN (100) and Fe_3_N (001). **g** 3D electron density difference distributions at the FeN/Fe_3_N interface. **h** The electrostatic potential of the FeN/Fe_3_N interface. **i** The electron localization function of the FeN/Fe_3_N interface. **j** 2D cross-section of electron density difference at the FeN/Fe_3_N interface. **k** The calculated work functions (W_f_) of FeN and Fe_3_N.

The FeN/Fe_3_N NPs were prepared via nitridation of an Fe salt that was pre-deposited on the commercial Ketjen (KJ) carbon support. In the process, Fe(NO_3_)_3_·9H_2_O urea and carbon support were mixed in water under ultrasonication, separated from the solution, washed with ethanol and deionized (DI) water, and dried under vacuum at 60 °C. The dried sample was then annealed under a flow of ammonia gas at different temperatures (300, 500, and 700 °C) to initiate iron nitride NP formation (see details in methods, and Supplementary Fig. 1). The NP crystal structure was analyzed by powder X-ray diffraction (XRD). Annealing at 300 °C led to the formation of FeN NPs with a cubic structure (space group F-43m, JCPDS No. 50 − 8017, *a* = *b* = *c* = 4.307 Å), annealing at 700 °C gave Fe_3_N NPs with a hexagonal structure (space group of P312, JCPDS No. 72 − 2125, *a* = *b* = 4.789 Å, *c* = 4.710 Å), while annealing at 500 °C yielded FeN/Fe_3_N NPs (Fig. 1a). The FeN/Fe_3_N, FeN, and Fe_3_N NPs are spherical and uniform, as shown in the scanning transmission electron microscopy (STEM) image (Fig. 1b and Supplementary Fig. 2). STEM mapping images display the similar Fe and N elements distribution across each FeN/Fe_3_N (Fig. 1c), FeN and Fe_3_N NP (Supplementary Fig. 2). The high-resolution TEM (HRTEM) image of the FeN/Fe_3_N NPs (Fig. 1d) shows clear FeN/Fe_3_N domains and interfaces with the lattice mismatch at 5.12% (detail in Methods). The detailed analysis of the FeN/Fe_3_N area by high resolution high-angle annular dark field aberration-corrected STEM (HAADF-STEM) reveals that the interface is created by the junction between cubic FeN and hexagonal Fe_3_N (Fig. 1e) with obvious atomic dislocation and two distinct diffraction spots at the junction (Supplementary Fig. 3). Away from the junction, the FeN (Supplementary Fig. 4) and Fe_3_N (Supplementary Fig. 5) regions are in single crystalline, showing no atomic dislocation. Additionally, the fast Fourier transform (FFT) images (Supplementary Fig. 6) of different regions in Fig.1e further confirm the FeN/Fe_3_N interface is formed with slightly distorted cubic and hexagonal structure. The N_2_ and CO_2_ adsorption ability of the Fe–N structures were measured by the Brunner–Emmet–Teller (BET) method under N_2_ and CO_2_ atmosphere (Supplementary Fig. 7). The FeN/Fe_3_N structure adsorbs more N_2_ (736.1 m^2^ g^−1^) than the FeN (314.5 m^2^ g^−1^) and Fe_3_N (586.0 m^2 ^g^−1^) ones, while the FeN structure adsorbs more CO_2_ (25.8 cm^2^ g^−1^) than the FeN/Fe_3_N (24.7 cm^2^ g^−1^) and Fe_3_N (11.2 cm^2 ^g^−1^) ones, suggesting that the FeN/Fe_3_N structure may be more porous, but it is the presence of the Fe–N_4_ coordination sites in the FeN structure that shows higher CO_2_ adsorption power.

We used the density functional theory (DFT) to further investigate the FeN/Fe_3_N structure (Fig. 1f–k). The (100) planes of FeN (pink) (interplanar distance 4.230 Å) and (001) planes of Fe_3_N (green) (interplanar distance 4.656 Å) match well to form the junction (Fig. 1f). We calculated electron density difference distributions^25^ between the FeN/Fe_3_N interface. As shown in Fig. 1g, the electron clouds around N and Fe in the FeN region (yellow) are much more enriched than that in the Fe_3_N region (blue), indicating the electron transfer from Fe_3_N to FeN. Due to this electron transfer, the FeN region becomes nucleophilic (Fig. 1h red and yellow region), while the Fe_3_N region is electrophilic (Fig. 1h blue region). The distribution of electrons across the interface structure was studied by 2D electron density distribution and electron location function, as shown in Fig. 1i, j. More electrons are localized over the FeN domain than over the Fe_3_N one. This is further supported by the calculated electrostatic potential^26^ (Fig. 1k) with the work function of FeN at 6.56 eV and Fe_3_N at 5.41 eV. Therefore, in the FeN/Fe_3_N interface structure, there exists a built-in electric field from Fe_3_N to FeN.

**Fig. 2:**
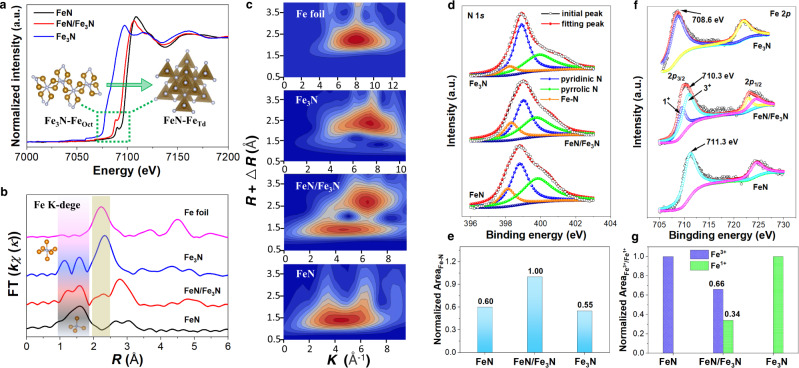
Fe-N NP coordination and electron structure characterization. **a** The normalized Fe K-edge EXAFS spectra. Inset: the local FeN structure showing tetrahedral coordination (right) and Fe_3_N structure showing octahedral coordination (left). **b** Fe K-edge FT-EXAFS in R space and **c** wavelet transforms for the k^3^-weighted EXAFS signals for FeN, FeN/Fe_3_N, Fe_3_N, and Fe foil. **d** The N 1 *s* XPS spectra, **e** the normalized area of Fe–N bond, **f** the Fe 2*p* XPS spectra, and **g** the normalized area of Fe^3+^/Fe^1+^ for FeN, FeN/Fe_3_N, and Fe_3_N, respectively.

The Fe K-edge of the extended X-ray absorption fine structure spectroscopy (EXAFS) of the Fe_3_N/FeN NPs show the increased energy of the fingerprint peak from Fe_3_N (7096.8 eV) to FeN (7108.5 eV) (Fig. 2a), indicating the Fe valence state change from +1 in Fe_3_N to +3 in FeN^27,28^. There is also a fingerprint peak (7106.4 eV) that is located in the middle of the Fe_3_N and FeN, implying that the Fe valence in the FeN/Fe_3_N interface region is between +1 and +3. The pre-edge peaks at 7089.7 and 7088.2 eV are from Fe−N_4_ with tetrahedral coordination (Fe_Td_, Insert in Fig. 2a right)^29,30^, not the octahedral coordination (Fe_Oct_, Insert in Fig. 2a left) in the Fe_3_N structure^31^. The Fourier transform k^3^-weighted EXAFS (FT-EXAFS) spectra in Fig. 2b (R space) show main peaks in the range from 1 to 3 Å. With more N binding to Fe, the Fe–Fe distance gets longer and longer (in the 2 − 3 Å range), but the Fe–N distance is shorter (in the 1–2 Å range), showing a double peak for the Fe_Oct_ − N (in the Fe_3_N structure) and a broad peak for the Fe_Td_ − N (in the FeN structure). The main peak at 2.33 Å in the Fe_3_N spectrum is close to the Fe−Fe bond (2.23 Å) measured from the Fe foil. Fittings of the FeN (Supplementary Fig. 8 and Supplementary Table 2) and Fe_3_N (Supplementary Fig. 9 and Supplementary Table 3) spectra suggest that Fe has a coordination number (CN) of 3.92 and 1.40 respectively. The FeN/Fe_3_N structure shows more complex peaks in the R-space with Fe−N_4_ peak at 1.60 Å and Fe–N_2_ peak at 2.33 Å. The corresponding fitting gives the CN of 3.60 for the Fe–N_4_ and 1.80 for the Fe–N_2_ structure (Supplementary Fig. 10 and Supplementary Table 4). The Fe–N structures were further analyzed by wavelet transform of the EXAFS^26^. As shown in Fig. 2c, both FeN and FeN/Fe_3_N have the K values centered around 4.8 Å^−1^, while those values from Fe_3_N and FeN/Fe_3_N locate at 6.5 (FeN/Fe_3_N) and 7.5 Å^−1^ (FeN), closer to 8.2 Å^−1^ observed from the Fe foil. All these analyses confirm that the Fe_3_N structure has more metal character than the FeN structure.

The Fe–N network structure was characterized by Raman spectroscopy (Supplementary Fig. 11). The same *D* and *G* peak position with slightly different I_*D*_/I_*G*_ values (1.26 for FeN/Fe_3_N, 1.15 for FeN, and 1.21 for Fe_3_N) suggest that more defects exist in the FeN/Fe_3_N structure^32^, which supports what is concluded from the BET N_2_-adsorption analysis. X-ray photoelectron spectroscopy (XPS) shows that the NP sample contains C (C 1 *s* spectra at 284.6 eV, Supplementary Fig. 12) and three different N’s (N 1 *s* spectra, Fig. 2d)^33^. The FeN/Fe_3_N interface structure has the largest Fe–N bonding area (Fig. 2e). Two spin-orbit doublets of Fe 2*p* can be found in Fe 2*p* spectra (Fig. 2f). The Fe 2*p*_3/2_ peaks shift from 711.3 eV (for FeN) to 708.6 eV (for Fe_3_N) while the peak for the FeN/Fe_3_N interface structure locates at 710.3 eV, indicating that the Fe valence state in the FeN/Fe_3_N structure is between FeN and Fe_3_N and electrons are drawn from Fe_3_N to FeN. Comparing with the normalized Fe−N bonding area for FeN and Fe_3_N, we calculated that the FeN/Fe_3_N interface structure contained 66% FeN and 34% Fe_3_N (Fig. 2g). The Fe binding environment was finally characterized by Mössbauer spectroscopy^34,35^, as shown in Supplementary Fig. 13. The fitting matches well with the experimental spectra, indicating that the FeN, FeN/Fe_3_N, and Fe_3_N structures have the Fe−N_4_, Fe−N_4_/Fe−N_2_, and Fe−N_2_ coordination, respectively.

**Fig. 3:**
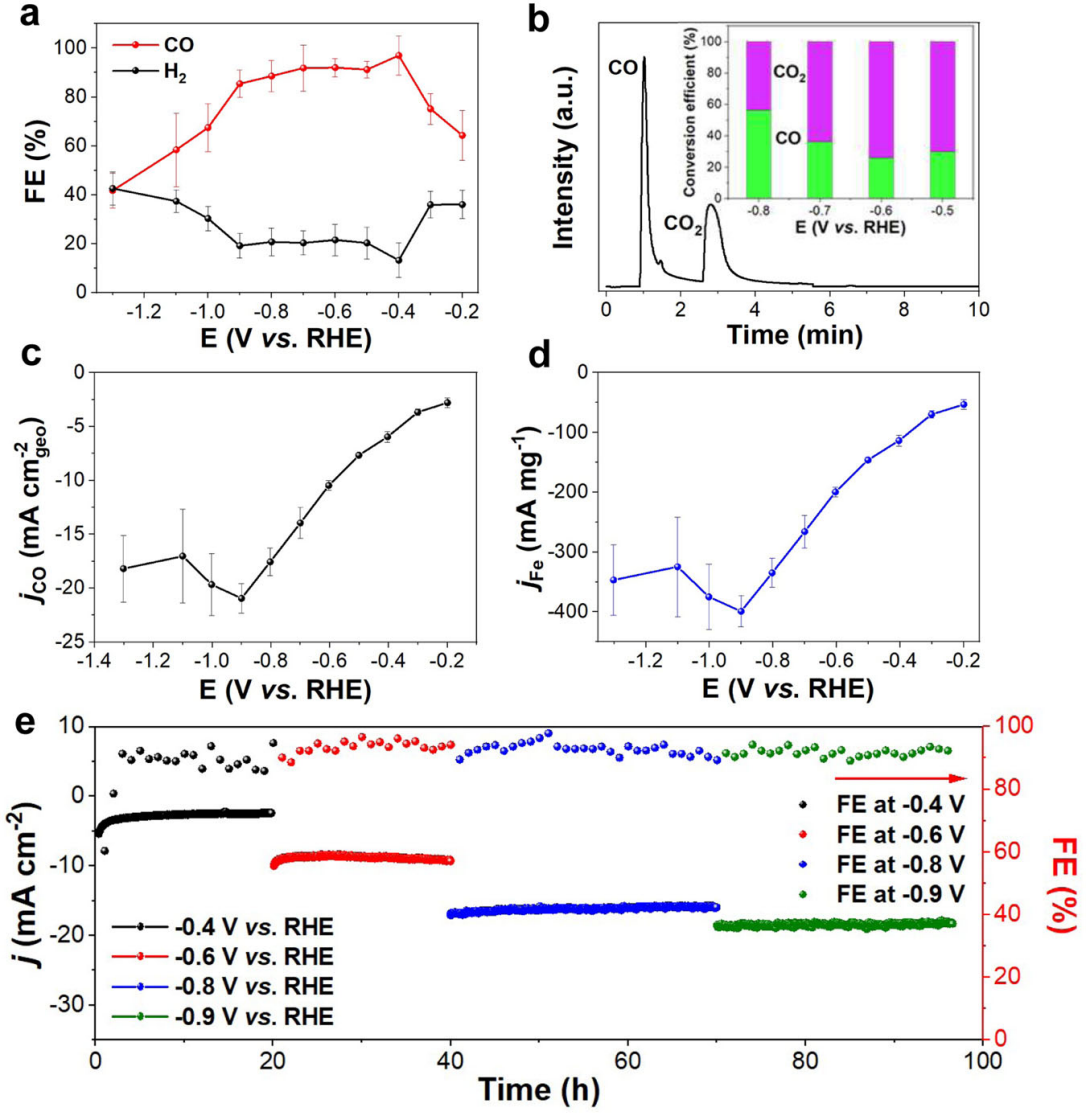
CO_2_RR performance of the FeN/Fe_3_N catalyst. **a** Reduction potential dependent FEs of CO and H_2_. **b** GC spectrum of the gas products upon a single pass CO_2_ conversion (Inset: reduction potential dependent CO_2_ conversion to CO). **c** CO partial current density and **d** corresponding mass activity. **e** CO_2_RR current density and CO FE changes at different reduction potentials over different electrolysis times. Error bars in **a**–**d** represent the standard deviation of three independent measurements.

The electrocatalytic performance of these Fe−N NP catalysts for CO_2_RR was evaluated by linear scan voltammetry (LSV, Supplementary Fig. 14) and cyclic voltammetry (CV, Supplementary Fig. 15) in an H-type cell filled with the Ar- and CO_2_-saturated 0.5 M KHCO_3_ electrolyte at room temperature^36,37^. The FeN/Fe_3_N structure is more responsive to the voltammetry tests, showing higher current density than either the FeN or Fe_3_N structure, suggesting that it initiates more CO_2_RR. Under a constant reduction potential, CO_2_RR products were characterized by gas chromatograph (GC) and nuclear magnetic resonance (NMR). In the current electrocatalytic reaction condition, the FeN/Fe_3_N structure is the most active catalyst for reducing CO_2_ to CO with the CO formation FE reaching 98% at −0.4 V and above 90% at −0.5 to −0.9 V (Fig. 3a). The H_2_ was another gas product (Supplementary Fig. 16) and no liquid products were detected (Supplementary Fig. 17). The FeN/Fe_3_N catalyst shows excellent CO selectivity compared with the recently reported catalysts (Supplementary Table 5). When the reduction potential more negative than −0.9 V, more HER was observed, which was further confirmed by DFT calculations that the H desorption energy was more than 0.9 eV on the FeN/Fe_3_N surface (Supplementary Fig. 18). This is also consistent with what was observed previously on *H binding energy increase at high potentials on Fe−N-based catalyst surfaces^24,38^. Based on the GC data from the closed reaction system, the single-pass CO_2_ conversion^39^ reached up to 56% at −0.8 V (Fig. 3b). The FeN/Fe_3_N catalyst is much more selective than N doped KJ carbon (CO FE is <20%), FeN (CO FE peaks at ~80% at −0.6 V) and Fe_3_N (CO FE peaks at ~70% at −0.6 V) (Supplementary Fig. 19), which indicates that the FeN/Fe_3_N interface structure is key to this CO selectivity enhancement. Its highest CO partial current density is 21 mA cm_geo_^−2^ (Fig. 3c) and the corresponding mass activity is 400 mA mg_Fe_^−1^ at −0.9 V, at which its turnover frequency (TOF, Supplementary Fig. 20) is 116 s^−1^ and turnover number (TON, Supplementary Fig. 20) is 99. The current density data were also normalized by geometric area of working electrode and CO_2_-BET area (Supplementary Fig. 21), all of which indicate the high catalytic performance of the FeN/Fe_3_N heterostructure. The FeN/Fe_3_N catalyst is superior to other representative Au, Ag, and M − N catalysts recently reported for catalyzing CO_2_RR to CO (Supplementary Table 5). The stability of the FeN/Fe_3_N catalyst was evaluated by time-dependent current change in 0.5 M KHCO_3_ at different potentials −0.4, −0.6, −0.8 and −0.9 V (Fig. 3e), which shows negligible decrease in both current density and CO FE in the 100 h testing period. It is worth to note that the CO FE stays above 90% during the tests, which is much better than the stability data obtained from other catalysts previously reported (Supplementary Table 5). We re-evaluated the FeN/Fe_3_N structure after the stability test. The XRD pattern (Supplementary Fig. 22a) still shows typical FeN and Fe_3_N peaks, the NP morphology is well-maintained in the TEM image (Supplementary Fig. 22b), and the interface structure is intact as evidenced by corresponding auto correlation and FFT images (Supplementary Fig. 22c). The XPS spectra of Fe 2*p* (Supplementary Fig. 23a) and N 1 *s* (Supplementary Fig. 23b), the EXAFS Fe K-edge spectra of the FeN/Fe_3_N (Supplementary Fig. 24a), the pre-edge peak in Fe K-edge (Supplementary Fig. 24a) and FT-EXAFS in R-space (Supplementary Fig. 24b) all indicate there is no obvious valence state change on Fe in the Fe−N structures, further confirming that the FeN/Fe_3_N heterostructure is well-preserved after the stability test.

**Fig. 4:**
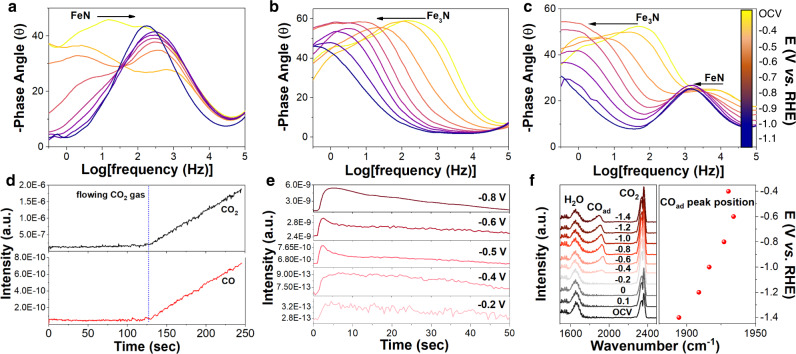
In situ spectroscopic studies of Fe-N catalysts and CO_2_RR. **a**–**c** Bode phase plots of FeN **a**, Fe_3_N **b**, and FeN/Fe_3_N **c** at different reduction potentials. In situ DEMS plots showing the increased presence of CO with increased flowing CO_2_ gas **d** and CO signal under different potentials **e** of the FeN/Fe_3_N-catalyzed CO_2_RR. **f** The in situ SEIR spectra of the FeN/Fe_3_N for CO_2_RR.

The evolution of the adsorbed CO_2_RR intermediates on Fe−N_4_, Fe−N_2_, and Fe−N_4_/Fe−N_2_ sites was studied by in situ electrochemical spectroscopies. The in situ electrochemical impedance spectroscopy (EIS)^40,41^ gives frequency-dependent changes of the Bode phase plots of the Fe−N-coordination sites, which is sensitive to the charge transfer and surface adsorption. The peaks of the FeN structure shift from middle frequency (10^0^ − 10^2^ Hz) to high-frequency (10^2^ − 10^4^ Hz) region (Fig. 4a) due to the low charge transport ability of the Fe−N_4_ coordination sites, while those of the Fe_3_N structure shift from high-frequency (10^2^ − 10^4^ Hz) to middle frequency region (10^0^ − 10^2^ Hz) (Fig. 4b) due to the poor surface adsorption of CO on the Fe–N_2_ coordination sites. In comparison, the FeN/Fe_3_N structure shows both FeN- and Fe_3_N-type Bode phase peaks, but these peaks shift to lower frequency (Fig. 4c) due to the optimized surface adsorption energy and enhanced charge transport ability from FeN_3_ to FeN.

We further conducted in situ differential electrochemical mass spectroscopy (DEMS) test to characterize products separated by the FeN/Fe_3_N-catalyzed CO_2_RR, from which the concentration of CO increases with the flowing CO_2_ gas (Fig. 4d), suggesting the detected CO is due to CO_2_RR. The CO signal in DEMS tests is enhanced from −0.2 to −0.8 V *vs*. RHE (Fig. 4e), indicating that the CO is the main product for CO_2_RR (Supplementary Fig. 25). We performed in situ spectra in the CO_2_-flowed 0.5 M KHCO_3_ electrolyte at various reduction potentials to characterize CO_2_ reduction intermediates. The *CO and *H competition was evidenced from in situ surface enhancement infrared spectroscopy (SEIR)^42,43^. According to the electrochemical Stark effect^44,45^, the vibrational frequencies of the intermediates adsorbed on an electrode usually shift negatively. As shown in Fig. 4f, there is no peak observed at ~1900 cm^−1^ from open circuit voltage (OCV) to −0.2 V. However, at −0.4 V, a small peak at ~1920 cm^−1^ starts to appear, and shows a negative shift with 49.66 cm^−1^ per voltage from −0.4 to −1.2 V, which attributes to the adsorbed CO* peak^46–49^. At more negative potentials, the intensity of the CO peak increases initially, but starts to drop after −1.2 V due to the strong competition of H* generated from the enhanced HER^50^. From the Raman spectra (Supplementary Fig. 26a), the electrolyte, CO_2_, CO, and H_2_O peaks can be found. The two peaks for HCO_3_^−^ at ~1010 cm^−1^ and CO_3_^2−^ at ~1060 cm^−1^ are found from the 0.5 M KHCO_3_ electrolyte^51^, while the ~1920 cm^−1^ peak has a negative shift from −0.4 to −0.8 V, which corresponds to the stretching vibration mode of the adsorbed CO^45^ (Supplementary Fig. 26b). In situ Raman spectra of CO_2_ adsorption peaks at ~2330 cm^−1^ for FeN (Supplementary Fig. 26c), Fe_3_N (Supplementary Fig. 26d), and FeN/Fe_3_N (Supplementary Fig. 26e) also intensify with enhanced voltage from OCV to −0.8 V vs. RHE (Supplementary Fig. 26f), demonstrating that the FeN/Fe_3_N heterostructure has an optimized interfacial adsorption ability.

**Fig. 5:**
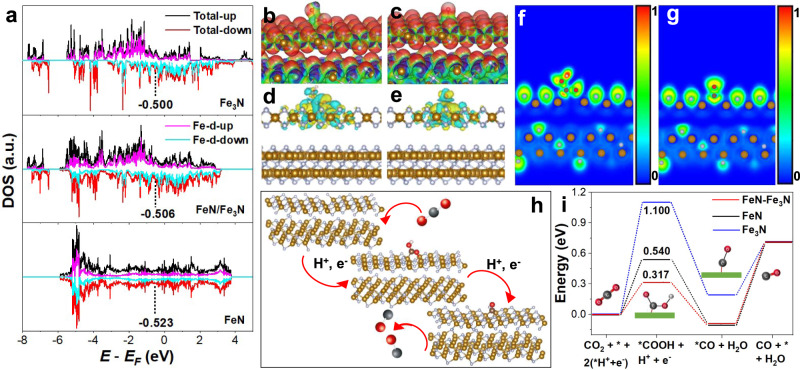
DFT calculations on the Fe−N catalyzed CO_2_RR. **a** The electronic density of states and d-band centers of Fe in the FeN, Fe_3_N, and FeN/Fe_3_N structures. The electrostatic potential for adsorption of COOH **b** and CO **c** on the FeN/Fe_3_N interface. 3D electron density difference distributions for adsorption of COOH **d** and CO **e** on the FeN/Fe_3_N interface. The electron localization for adsorption of COOH **f** and CO **g** on the FeN/Fe_3_N interface. **h** Schematic reaction CO_2_RR to CO pathway projected on the FeN/Fe_3_N surface. **i** The calculated Gibbs free energy diagrams for CO_2_RR on the FeN, Fe_3_N, and FeN/Fe_3_N surfaces.

The formation of CO from CO_2_RR is generally believed to follow two proton-coupled electron-transfer steps to form COOH*, and then CO* that is desorbed from the catalyst surface^52–54^. In this process, the formation of COOH* usually is a potential-limiting step due to the high activation energy of CO_2_ molecule. To understand the Fe−N catalysis better, we used DFT to calculate the related activation energy on the catalyst surfaces. We compared the electronic density of states (DOS) and d-band center of Fe_3_N, FeN, and FeN/Fe_3_N structures^55–57^. As shown in Fig. 5a, the d-band center of the FeN/Fe_3_N heterostructure is at −0.506 eV, while that of FeN is at −0.523 eV and Fe_3_N at −0.500 eV, suggesting Fe_3_N is beneficial to improve the overall d-band center shift to the Fermi level (E_F_). Additionally, the d-band center of pure FeN (100), pure Fe_3_N (001), FeN cell, and Fe_3_N cell is −0.591, −0.503, −0.647, and −0.502 eV (Supplementary Fig. 27), further demonstrating Fe_3_N with rich electron is key for built-in electric field from Fe_3_N to FeN. As a result, the intensity of DOS (Supplementary Fig. 28), and the integral DOS (IDOS) (Supplementary Fig. 29) of the FeN/Fe_3_N structure is higher than that of the FeN or Fe_3_N structure, suggesting that electrons are more populated at E_F_ level on the FeN/Fe_3_N surface for effective binding of reaction intermediates. The FeN/Fe_3_N surface potential change upon binding to COOH and CO was calculated by electrostatic potentials and shown as colored contours in Fig. 5b (for *COOH) and Fig. 5c (for *CO), which shows that electron transfer causes a decrease in the potential of CO and COOH (red means lower electrostatic potential and blue means higher electrostatic potential). The corresponding 3D electron density distributions on the FeN/Fe_3_N (Fig. 5d, e), FeN (Supplementary Fig. 30c, d), and Fe_3_N (Supplementary Fig. 31c, d) surfaces upon their binding to COOH and CO suggest that electrons are transferred from catalyst to COOH/CO. The electron localization of COOH and CO on the FeN, Fe_3_N, and FeN/Fe_3_N surface calculated by electron localization function is about 0.8, 0.7, 0.8 (Supplementary Fig. 30e, Supplementary Fig. 31e and Fig. 5f) for COOH and 0.7, 0.7, 0.7 (Supplementary Fig. 30f, Supplementary Fig. 31f and Fig. 5g) for CO, indicating that COOH and CO interact covalently with the catalyst surface^58–60^. Following the common CO_2_RR reaction pathway specifically on the FeN/Fe_3_N surface (Fig. 5h), we calculated the Gibbs free energy (ΔG) for the potential-limiting step (CO_2ad_ to *COOH) on the FeN/Fe_3_N structure to be 0.317 eV (Fig. 5i), much lower than that on the FeN (0.540 eV) and Fe_3_N (1.10 eV) in the heterostructure. We also calculated the CO_2_RR performance on the other side of FeN/Fe_3_N heterostructure on the interface Fe_3_N (001) (Supplementary Fig. 32a) and other side of Fe_3_N (001) (Supplementary Fig. 32b). The ΔG for CO_2_ to COOH is 0.985 eV on the interface Fe_3_N (001), ΔG for *CO on other side of Fe_3_N (001) is −2.566 eV, which is too stable for the CO to dissociate from the plane. ΔG for CO_2_ to COOH is about 0.360 and 1.08 eV on the pure FeN (100) (Supplementary Fig. 32c) and pure Fe_3_N (001) (Supplementary Fig. 32d), indicating that CO_2_ adsorption and activation on the FeN (100) is more favorable for CO_2_RR. We further constructed the FeN (110)/Fe_3_N (001) heterostructure to reveal the role of the FeN (110) plays on catalyzing CO_2_RR. The electron cloud around N and Fe in the Fe_3_N region (yellow) is much more enriched than that in the FeN region (blue) (Supplementary Fig. 33a), while is also supported by the 2D electron localization function (Supplementary Fig. 33b). These indicate that the FeN (110)/Fe_3_N (001) structure has no electron transport across interface and is not active for CO_2_RR. Then, the ΔG for CO_2ad_ to *COOH on the FeN (110)/Fe_3_N (001) structure is 1.189 eV (Supplementary Fig. 33c) much larger than that of FeN (100)/Fe_3_N (001) heterostructure, further demonstrating that FeN (100)/Fe_3_N (001) heterostructure is superior for CO_2_RR. Our calculations support what we concluded from both structure and catalysis analysis of the Fe−N catalysts that the FeN (100)/Fe_3_N (001) interface with the built-in electric field has abundant electrons closer to E_F_ for CO_2_ adsorption and activation, showing much enhanced catalysis for CO_2_RR to CO.

## Discussion

In summary, we have reported a process for synthesizing heterostructured FeN/Fe_3_N NPs with the NP surface populated with Fe–N_4_/Fe–N_2_ interfaces. Electron polarization from Fe_3_N (Fe−N_2_ site) to FeN (Fe−N_4_ site) builds the desired electric field across the FeN/Fe_3_N interface, enhancing its power to adsorb CO_2_. In the electrochemical reduction condition, this FeN/Fe_3_N structure has an excellent mass and electron transfer ability in catalyzing CO_2_RR to CO with high FE (98% at −0.4 V) and much improved stability in the 100 h electrolysis period (FE > 90% in the broad −0.4 to −0.9 V potential range). The catalyst surface adsorption of CO_2_ and its activation intermediates is further characterized by various in situ spectroscopy techniques (EIS, DEMS, IR and Raman) from which the adsorbed CO* is identified. DFT calculations suggest that the FeN/Fe_3_N structure has a d-band center that is closer to its E_F_ level where there are abundant electrons for binding to reaction intermediates (CO_2ad_, COOH*, CO*). This strong interaction reduces the ΔG of the CO_2ad_ to COOH* conversion and provides a necessary force to lower the reduction overpotential and improve the catalytic efficiency. Our work demonstrates that creating a polarized field on the NP surface is a promising approach to the design of highly active catalysts for electrochemical CO_2_RR.

## Methods

### Synthesis of Fe−N based precursor

Firstly, the commercial Ketjen (KJ) carbon (0.1 g) was suspended in water (H_2_O, 50 mL) by ultrasound. Then, a mixture of iron (III) nitrate hexahydrate (Fe(NO_3_)_3_•9H_2_O, 0.01 mol, 4.04 g) and urea (CH_4_N_2_O, 0.1 mol, 6.00 g) was added into the carbon suspension and continued ultrasound for 8 h. Finally, the precipitate was separated by centrifuge (6000 rpm (4830 × *g*), 5 min) and washed with water and ethanol for three times and dried under vacuum at 60 °C for 6 h to give the Fe−N based precursor.

### Synthesis of FeN, FeN/Fe_3_N, and Fe_3_N nanosparticles

100 mg Fe−N precursor was placed in crucible and put in the tube furnace (Supplementary Fig. 1). Then the tube was heated to 300, 500 or 700 °C with a rate of 5 °C min^−1^ for 2 h under the flowing NH_3_ atmosphere. Finally, the system was cooled to room temperature under a flowing NH_3_ gas. In this process, FeN, FeN/Fe_3_N and Fe_3_N were obtained at 300 °C, 500 °C, and 700 °C, respectively^33^.

### Catalyst characterization

X-ray diffraction (XRD) experiments were conducted from 20° to 90° on an X’Pert ProX-ray diffractometer with Cu Ka radiation (λ = 0.1542 nm) under a voltage of 40 kV and a current of 40 mA. Sample compositions were determined by ICP-AES (HITACHI P-4010, Japan). Product morphology was imaged by field-emission scanning electron microscopy (FESEM, Zeiss) at an acceleration voltage of 5 kV. All samples were coated with a thin layer of gold prior to FESEM observations. More detailed product morphology and structure were analyzed by transmission electron microscopy (TEM) and high-resolution transmission electron microscopy (HRTEM) under an acceleration voltage of 200 kV with a JEOL JEM 2100 TEM. Atomic-scale STEM images were recorded on a probe aberration-corrected STEM (Cubed Titan G2 60-300, FEI, USA) operated at 300 kV. Product surface composition was analyzed by X-ray photoelectron spectroscopy (XPS) with a VG ESCALAB 220I-XL device. All XPS spectra were corrected using C1*s* line at 284.6 eV. The absorption spectra of Fe K-edge were collected in transmission mode using a Si (111) double-crystal monochromator at the BLW141 station of the Beijing Synchrotron Radiation Facility (BSRF). Transmission ^57^Fe Mössbauer spectra were recorded by using a conventional spectrometer working in constant acceleration mode. A 50 mCi of ^57^Co embedded in a Rh matrix moving at room temperature was used as the γ-ray source. The absorber was prepared with a surface density of ~8 mg cm^−2^ natural iron. The drive velocity was calibrated with sodium nitroprusside at room temperature and all the isomer shifts quoted in this work are relative to that of the α-Fe.

### Lattice mismatch calculation

The lattice mismatch for the heterostructure can be calculated by lattice of different domains, as shown below:

1 $$\delta=(2\times|{{{{{{\rm{a}}}}}}}_{1}-{{{{{{\rm{a}}}}}}}_{2}|)/({{{{{{\rm{a}}}}}}}_{1}+{{{{{{\rm{a}}}}}}}_{2})$$

where *δ* is lattice mismatch, a_1_ and a_2_ are the lattice parameters of different domains. We define that two lattices are well-matched when *δ* is *<*5%, semi-matched if *δ* is between 5% and 25%, and totally mismatched if *δ* is >25%. The *δ* value for the FeN/Fe_3_N heterostructure is 5.12% (*δ*_FeN/Fe3N_ = (2 × |a_FeN_ – a_Fe3N_ | )/(a_FeN_ + a_Fe3N_) = (2 × |0.19 – 0.20 | )/(0.19 + 0.20) = 5.12%), therefore, the FeN and Fe_3_N lattices are well-matched, which is favorable for electron transfer at the interface.

### Electrochemical test for CO_2_RR

Electrochemical measurements were carried out at room temperature using the three-electrode system directly connected to a CHI 760 E Electrochemical Workstation (CHI Instruments, Shanghai Chenhua Instrument Corp., China). The CHI workstation was used to conduct CO_2_ reduction experiments in aqueous 0.5 M KHCO_3_ (pH = 6.8 when saturated with CO_2_, pH = 8.3 when saturated with Ar). A platinum wire was used as counter electrode. All potentials were measured against an Ag/AgCl reference electrode (4.0 M KCl, Pine instrument) and were calibrated against a reversible hydrogen electrode (RHE). The experiments were performed in a gas-tight cell with two-compartments separated by an anion exchange membrane (Nafion® 212). Each compartment contained 10 mL electrolyte with approximately 10 mL headspace^6,37^.

### Product analysis

CO_2_ gas was delivered at an average rate of 30 mL min^−1^ (at room temperature and ambient pressure) and routed directly into the gas sampling loop of a gas chromatograph (Agilent 7890 A). The gas phase composition was analyzed by GC every 30 min. The GC analysis was set up to split the gas sample into two aliquots whereof one aliquot was routed through a packed MoleSieve 5 A column and a packed HP-PLOT Q column before passing a thermal conductivity detector (TCD) for H_2_ quantification. Argon (Corp Brother, 99.9999%) and Helium (Corp Brother, 99.9999%) were employed as carrier or make-up gases respectively. The second aliquot was routed through a packed HP-PLOT Q + PT column equipped with a flame ionization detector (FID) for analyzing CO and C_1_ to C_3_ hydrocarbons. The GC was calibrated using commercially available calibration standards from JJS Technical Services. ^1^H-NMR spectroscopy was employed to characterize and quantify CO_2_ reduction products at the end of the experiments. ^1^H-NMR spectra were recorded on Bruker DRX 400/600 Avance. A total of 0.5 ml electrolyte and 0.1 ml D_2_O (content 0.05 μL dimethyl sulfoxide (DMSO), as inner standard) were mixed together to get the NMR signal.

### Theoretical calculations

The first-principles density functional theory (DFT) calculations were performed within the generalized gradient approximation (GGA) based on via Vienna abinitio simulation package (VASP)^61–63^. We constructed FeN/Fe_3_N interface structure, FeN and Fe_3_N surface model with periodicity in the x and y directions and the depth of the vacuum layer is greater than 20 Å in order to prevent self-interactions. In addition, the bottom two stoichiometric layers were need to be fixed while the top layer and adsorbates (CO*, COOH*) were allowed to relax in the optimization of the FeN/Fe_3_N and Fe_3_N, for FeN, only one stoichiometric layer needs to be fixed. The VASP was employed to perform all DFT calculations within the functional as proposed by Perdew-Burke-Ernzerh (PBE)^61,64^ of interactions is represented using the projector augmented wave (PAW)^61,65,66^ potential. The Kohn-Sham one-electron valence states were expanded on the basis of plane waves with a cutoff energy of 400 eV in the in the process of structure optimization. A geometry optimization was considered convergent when the electronic energy and Hellmann-Feynman forces convergence criterion was smaller than 10^−5^ eV and 0.03 eV Å^−1^, respectively. The K-point of 3 × 1 × 1 was used for the optimization of the FeN/Fe_3_N, FeN and Fe_3_N surface, and the corresponding adsorption structure (CO*, COOH*). Subsequently, we calculated the charge density, electron localization function (ELF), work function and electrostatic potential in the self-consistent process. The electronic energy was considered self-consistent while the energy variation was smaller than 10^−5^ eV. The value of K-point, cutoff energy is the same as the process of structure optimization. After the self-consistent calculation converges, the charge density distribution, electron localization, electrostatic potential and work function can be obtained, the self-consistent structure is used as the input file, and the wave function and charge density file are read to obtain the density of states of the heterojunction, FeN and Fe_3_N.

The d-band center was used to understand the adsorption capacity of the different catalyst (FeN/Fe_3_N, FeN and Fe_3_N) surface for CO_2_. The calculation formula of d-band center:

2 $${\varepsilon }_{d}=\frac{{\int }_{-\infty }^{\infty }{n}_{d}(\varepsilon )\varepsilon d\varepsilon }{{\int }_{-\infty }^{\infty }{n}_{d}(\varepsilon )d\varepsilon }$$

where *nd(ɛ)* represents the density of states and *ɛ* represents the energy of state.

The Gibbs free energy also was calculated in this study. The zero-point energy (ZPE) correction was performed referring to the approaches previously reported. In the DFT process, we calculated the Gibbs free energy according to the equations as follow:^61^

3 $${G}^{0}={E}_{{DFT}}+{ZPE}-T{S}^{0}$$

Where *G*^*0*^ is the Gibbs free energy, *E*_*DFT*_ is total free energy, *ZPE* is the vibration energy; *TS*^*0*^ is the entropy change (T = 298.15 K).

### TOF and TON calculation for FeN/Fe_3_N catalyst

The turnover frequency (TOF) was calculated by the following formula:^67^

4 $${{{{{\rm{TOF}}}}}}=\frac{j\times {{{{{\rm{S}}}}}}\times {{{{{\rm{FE}}}}}}}{2\times {{{{{\rm{n}}}}}}\times {{{{{\rm{F}}}}}}}$$

where *j* (mA cm^−2^) is the current density at −0.4 V, *S* is the surface area of the working electrode, *FE* is the Faraday efficiency of CO, the number 2 means 2 electrons mol^–1^ of CO, *F* is Faraday’s constant (96485.3 C mol^–1^), and *n* is the moles of coated Fe atoms on the electrode. The Fe content of catalysts was measured by the ICP-AES measurement.

Similarly, the turnover number (TON) of the catalyst was calculated based on:

5 $${{{{{\rm{TON}}}}}}={{{{{\rm{TOF}}}}}}\times {{{{{\rm{FE}}}}}}\times 0.01$$

## Supplementary information

Supplementary Information

## Data Availability

All data supporting the findings of this study are available in the article and its Supplementary Information. Source data for the following figures are provided with this paper. Figure 1a, k, Fig. 2a–g, Fig. 3a–e, Fig. 4a–f, Fig. 5a, i, Fig. S7a–f, Fig. S8a and b, Fig. S9a and b, Fig. S10a and b, Fig. S11, Fig. S12, Fig. S13a–c, Fig. S14, Fig. S15a and b, Fig. S16, Fig. S17, Fig. S18, Fig. S19a–c, Fig. S20, Fig. S21a and b, Fig. S22a, Fig. S23a, b, Fig. S24a, b, Fig. S25, Fig. S26a–f, Fig. S27a–d, Fig. S28, Fig. S29, Fig. S32a–d, Fig. S33c. Source data are provided with this paper.

## Source data

Source File
