# Supplementary material for: The built-in electric field across FeN/Fe3N interface for efficient electrochemical reduction of CO2 to CO
Source: Nat Commun. 2023 Mar 28;14:1724. doi: 10.1038/s41467-023-37360-9 (PMC10050184; doi:10.1038/s41467-023-37360-9)
Supplement: Supplementary file 1 — Supplementary Information [file 41467_2023_37360_MOESM1_ESM.pdf]

The Built-in Electric Field across FeN/Fe_3N Interface for Efficient Electrochemical Reduction of CO_2 to CO

Jie Yin,[1,†]* Jing Jin,[1,†] Zhouyang Yin,[2,†] Liu Zhu,[3,†] Xin Du,[4]* Yong Peng,[3] Pinxian Xi,[1]* Chun-Hua Yan,[1,5] Shouheng Sun[2]*

[1]State Key Laboratory of Applied Organic Chemistry, College of Chemistry and Chemical Engineering, Lanzhou University, Lanzhou 730000, China.

[2]Department of Chemistry, Brown University, Providence, Rhode Island 02912, United States.

[3]Electron Microscopy Centre of Lanzhou University, Lanzhou University, Lanzhou 730000, China.

[4]College of Chemistry, Zhengzhou University, Zhengzhou 450001, China.

[5]State Key Laboratory of Rare Earth Materials Chemistry and Applications, Peking University, Beijing 100871, China

*Corresponding Author. Email: yinj@lzu.edu.cn; dux@zzu.edu.cn; xipx@lzu.edu.cn; ssun@brown.edu.

[†]These authors contributed equally to this work.

Supplementary Table 1 | CO_2 reduction to CO by some representative catalysts.

Catalysts	Electrolyte	FE_{CO}	Potential (V vs.RHE)	j_{CO} (mA cm^{-2})	single-pass conversion (%)	Stability (h)	Ref
In_A/NC	0.5 M [Bmim]PF_6/ MeCN	97.2%	−2.1 V vs Ag/Ag	39.4	−	24	1
Ni/Fe-C-N	0.5 M $KHCO_3$	98%	−0.7	7.4	−	30	2
Cu/SnO_2	0.5 M $KHCO_3$	93%	−0.7	4.6	−	−	3
Cu-In alloy	0.1 M $KHCO_3$	95%	−0.6	−	−	7	4
Fe^{3+}−N−C	0.5 M $KHCO_3$	94%	−0.45	94	−	12	5
TPY-MOL-CoPP	0.1 M $NaHCO_3$	92.2%	−0.86	1314	−	−	6
Pd@Pd_3Au_7 NCs	0.1 M $KHCO_3$	94%/100%	−0.5/−0.6 to −0.9	18.79	−	8	7
Porphyrin ligand P1 capped Au NPs	0.5 M $KHCO_3$	93%	−0.45	2	−	72	8
Ni-NC(AHP)	0.5 M $KHCO_3$	84.1%/100%	−0.6/−0.7 to −1.0	14.5	−	12	9
FeN/Fe_3N	0.5 M $KHCO_3$	98%	−0.4	21	56%	100	This work

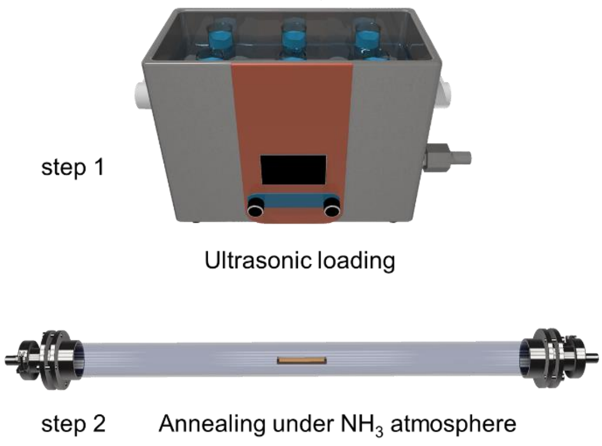

Supplementary Figure 1 | Schematic illustration of the synthetic system.

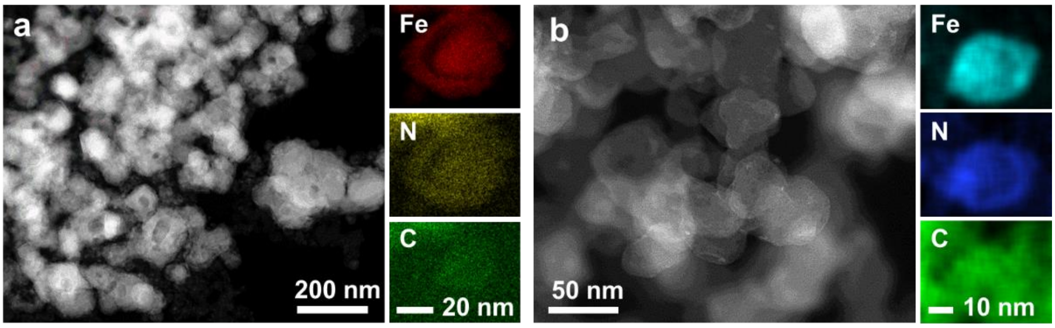

Supplementary Figure 2 | STEM images and elements mapping of (**a**) FeN and (**b**) Fe_3N NPs.

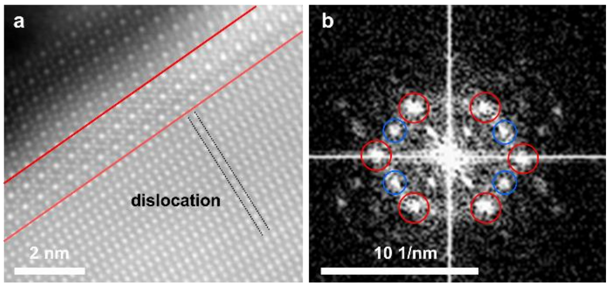

Supplementary Figure 3 | **a**, the atomic dislocation at the FeN/Fe_3N interface observed in HAADF-STEM image. **b**, the corresponding electron diffraction of red line area in (**a**).

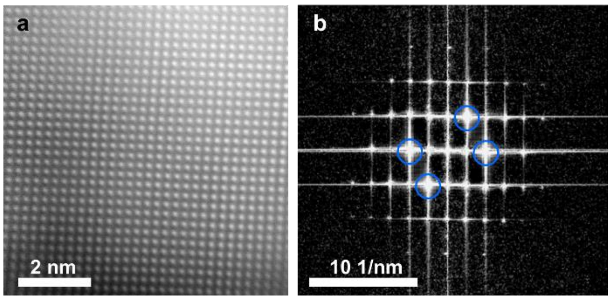

Supplementary Figure 4 | HAADF-STEM image (**a**) and corresponding electron diffraction (**b**) of FeN, showing the cubic atomic arrangement.

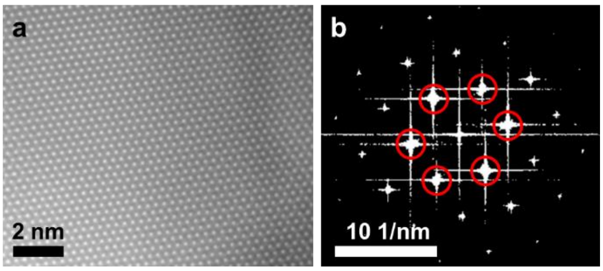

Supplementary Figure 5 | HAADF-STEM image (**a**) and corresponding electron diffraction (**b**) of Fe_3N, showing the hexagonal atomic arrangement.

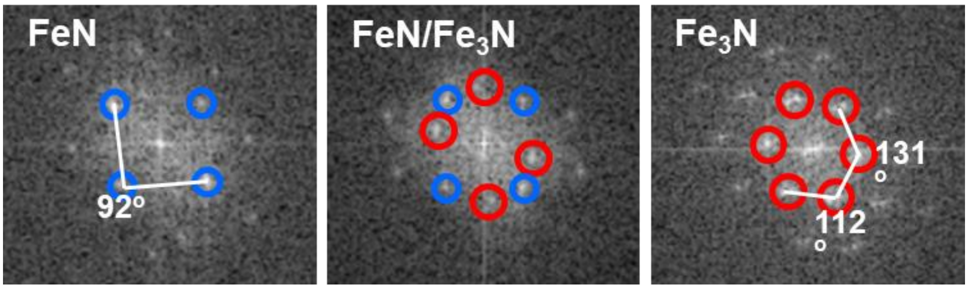

Supplementary Figure 6 | Corresponding FFT images with angle between the diffraction spots of **Fig.1e** for FeN (distorted cubic), Fe_3N (distorted hexagonal), and FeN/Fe_3N heterostructure.

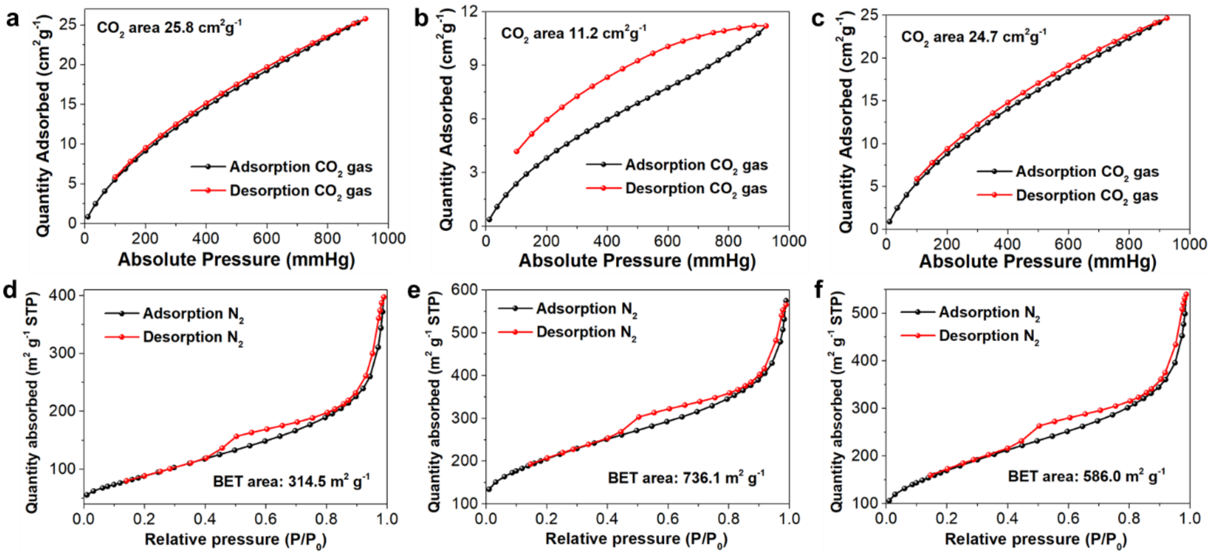

Supplementary Figure 7 | Surface area of Fe-N catalysts measured by CO_2 adsorption of (**a**) FeN, (**b**) FeN/Fe_3N, and (**c**) FeN_3, as well as by N_2 adsorption of (**d**) FeN, (**e**) FeN/Fe_3N, and (**f**) FeN_3.

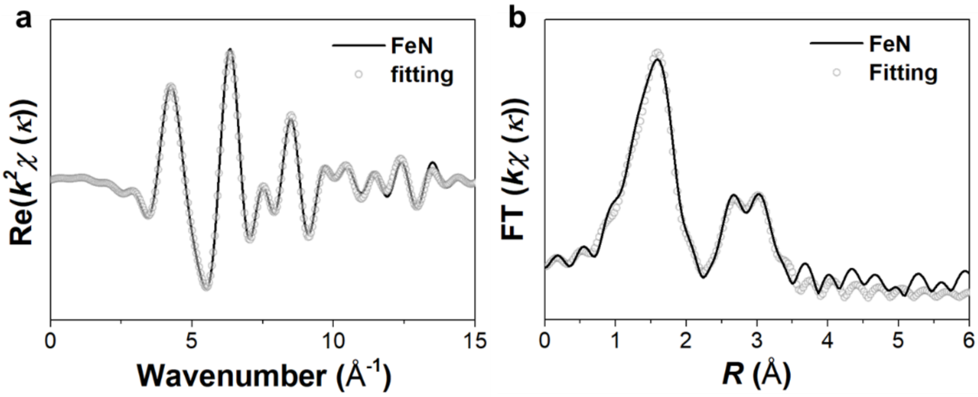

Supplementary Figure 8 | The fitting curve of $k^2\chi(k)$ oscillations (**a**) and k^3-weighted (**b**) EXAFS spectra of FeN.

Supplementary Table 2 | EXAFS fitting results at Fe K−edge for FeN

FeN	Scattering pair								
Fe_{Td}	Fe−N_1			Fe−Fe			Fe−N_2		
	CNs	R	σ^2	CNs	R	σ^2	CNs	R	σ^2
	3.92	2.02377	0.01255	0.864	3.07966	0.00597	2.56	3.38811	0.00076

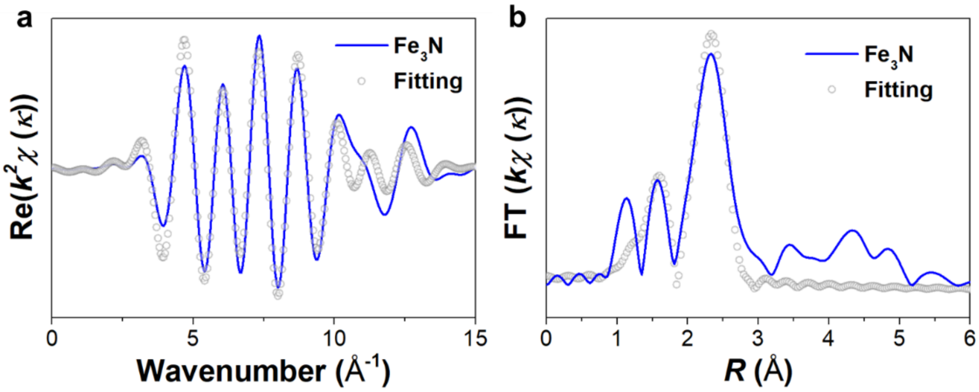

Supplementary Figure 9 | The fitting curve of $k^2\chi(k)$ oscillations (**a**) and k^3-weighted (**b**) EXAFS spectra of Fe_3N.

Supplementary Table 3 | EXAFS fitting results at Fe K−edge for Fe_3N

Fe_3N	Scattering pair					
Fe_{Oct}	Fe−N_1			Fe−Fe		
	CNs	R	σ^2	CNs	R	σ^2
	1.40	1.96412	0.00181	4.20	2.69055	0.00950

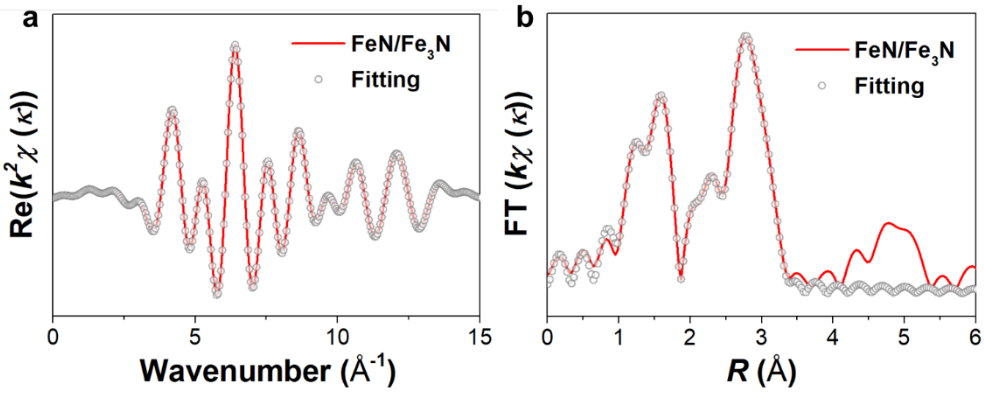

Supplementary Figure 10 | The fitting curve of $k^2\chi(k)$ oscillations (**a**) and k^3-weighted (**b**) EXAFS spectra of FeN/Fe_3N.

Supplementary Table 4 | EXAFS fitting results at Fe K−edge for FeN/Fe_3N

FeN/Fe_3N	Scattering pair								
Fe_{Td}	Fe−N_1			Fe−Fe			Fe−N_2		
	CNs	R	σ^2	CNs	R	σ^2	CNs	R	σ^2
	3.60	1.94861	0.01222	1.08	3.00395	0.01211	2.16	3.30235	0.14229
Fe_{Oct}	Fe−N_1				Fe−Fe				
	CNs	R	σ^2		CNs		R	σ^2	
	1.80	2.01267	0.01067		5.40		2.86249	0.00979	

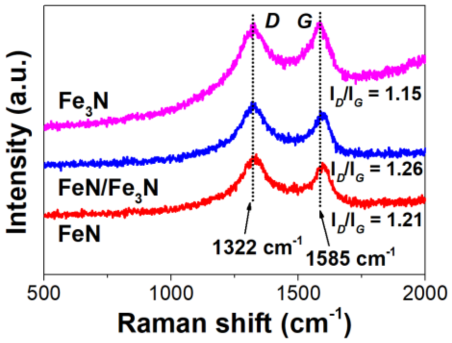

Supplementary Figure 11 | Raman spectra of FeN, FeN/Fe_3N, and Fe_3N.

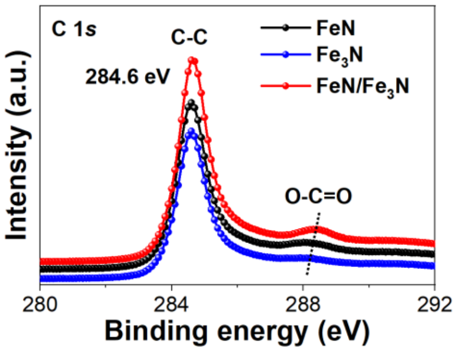

Supplementary Figure 12 | C 1s spectra of FeN, FeN/Fe_3N, and Fe_3N.

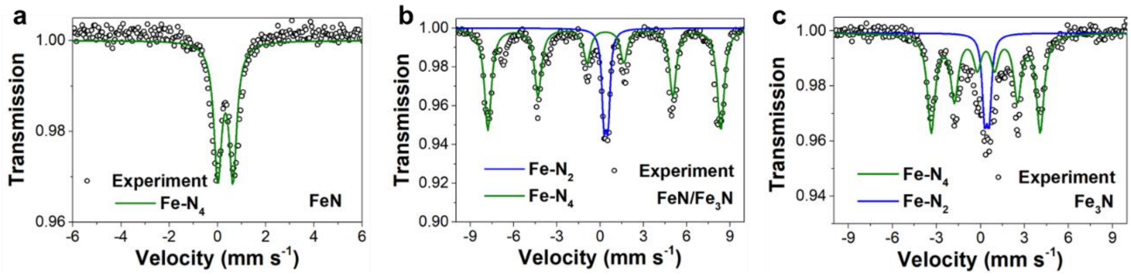

Supplementary Figure 13 | The fitting curve of Mössbauer spectra of (**a**) FeN, (**b**) FeN/Fe_3N, and (**c**) Fe_3N.

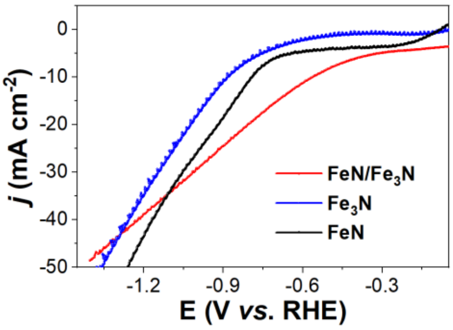

Supplementary Figure 14 | LSV curves of FeN, Fe_3N, and FeN/Fe_3N in the CO_2-saturated 0.5 M $KHCO_3$ electrolyte at room temperature.

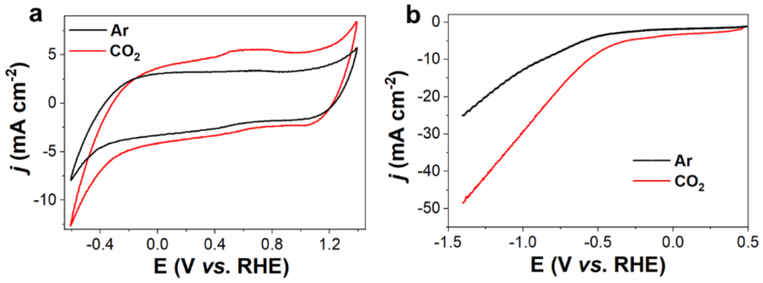

Supplementary Figure 15 | (**a**) CV and (**b**) LSV curves of FeN/Fe_3N in the CO_2/Ar-saturated 0.5 M $KHCO_3$ electrolyte.

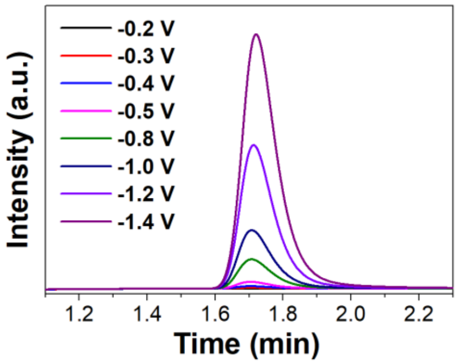

Supplementary Figure 16 | H_2 peaks measured by GC under different potentials.

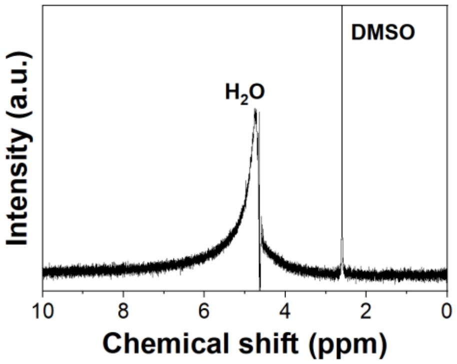

Supplementary Figure 17 | NMR spectrum of the product separated from FeN/Fe_3N-catalyzed CO_2RR in the CO_2-saturated 0.5 M $KHCO_3$ electrolyte.

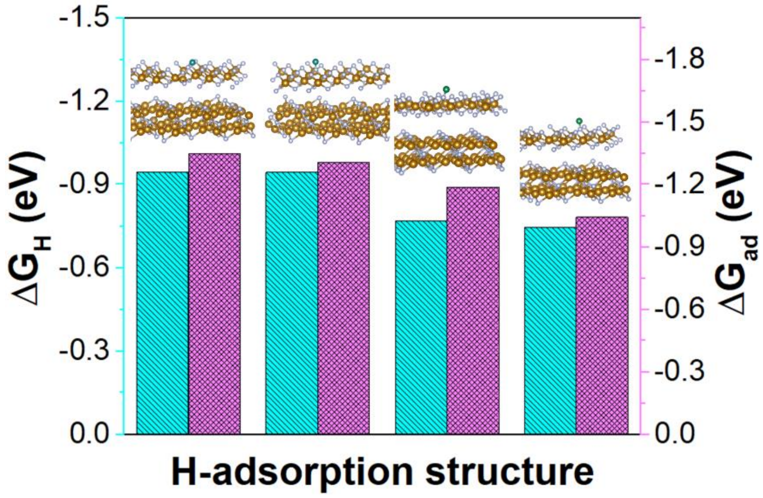

Supplementary Figure 18 | The energy required for ΔG_H and H desorption on different FeN/Fe_3N heterostructures.

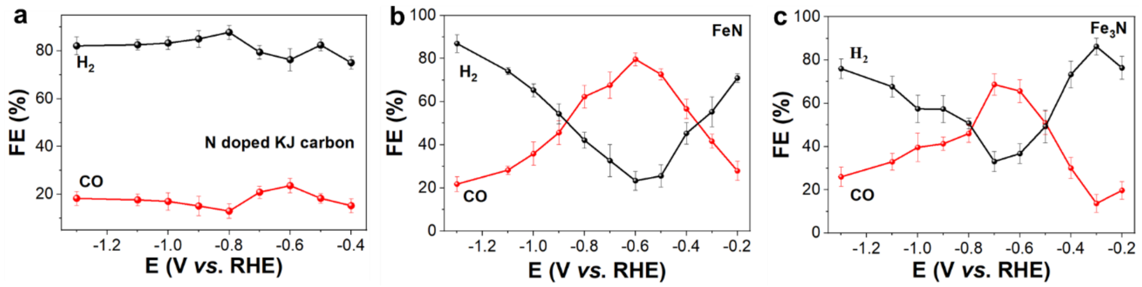

Supplementary Figure 19 | FE of CO and H_2 obtained from (**a**) N doped KJ carbon, (**b**) FeN and (**c**) Fe_3N catalysed CO_2RR in the CO_2-saturated 0.5 M $KHCO_3$ electrolyte. Error bars in (**a**), (**b**) and (**c**) represent the standard deviation of three independent measurements.

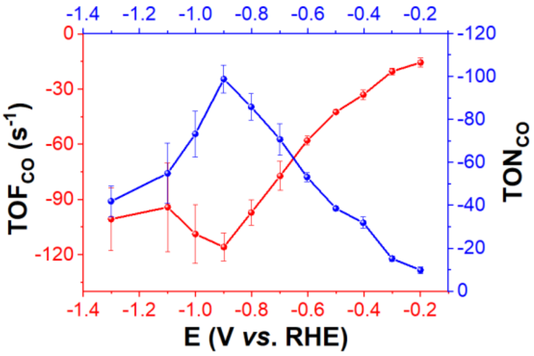

Supplementary Figure 20 | TOF and TON of FeN/Fe_3N for CO_2RR. Error bars represent the standard deviation of three independent measurements.

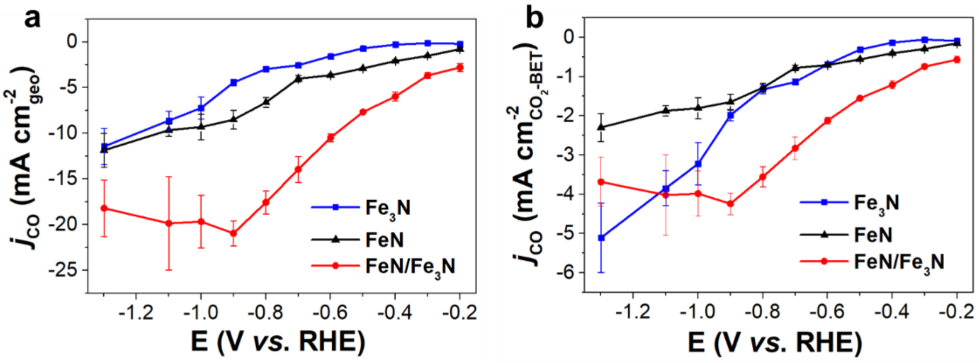

Supplementary Figure 21 | The efficient CO current density calculated by geometric area of working electrode (**a**) and CO_2-BET area (**b**) of FeN, Fe_3N, and FeN/Fe_3N. Error bars in (**a**) and (**b**) represent the standard deviation of three independent measurements.

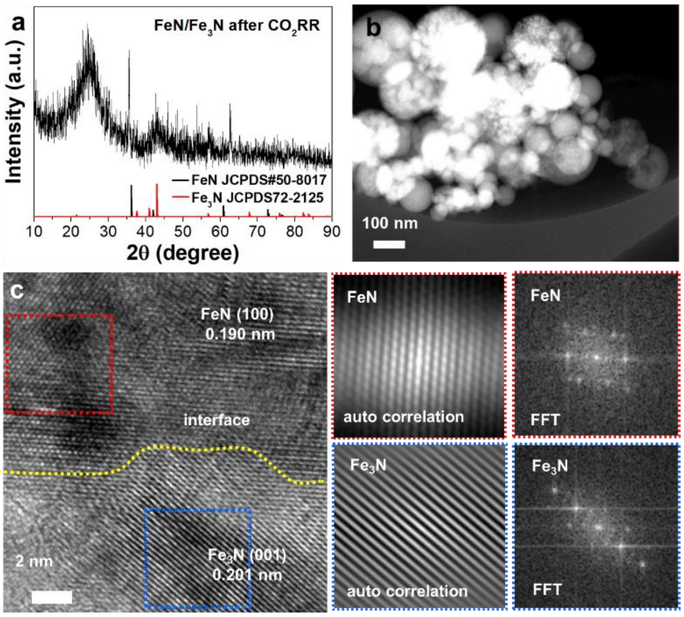

Supplementary Figure 22 | (**a**) XRD, (**b**) TEM, (**c**) HRTEM and corresponding auto correlation and FFT images of FeN/Fe_3N NPs after CO_2RR.

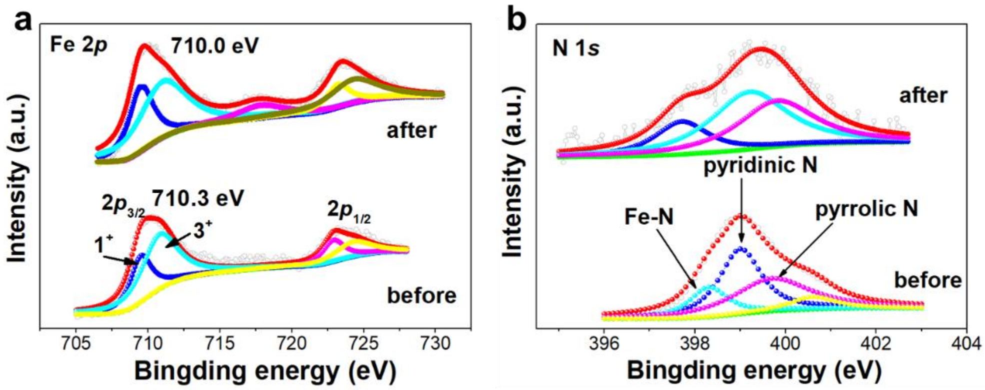

Supplementary Figure 23 | XPS spectra of (**a**) Fe 2*p* and (**b**) N 1*s* of FeN/Fe_3N after CO_2RR.

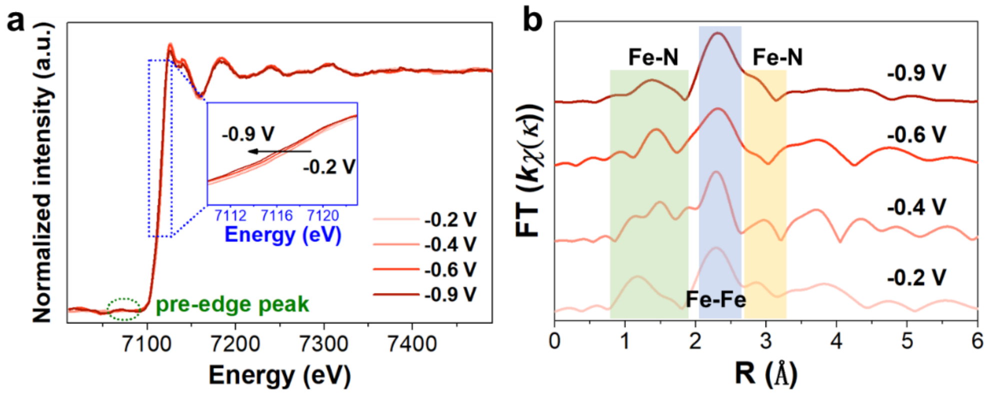

Supplementary Figure 24 | The normalized Fe K-edge EXAFS spectra (**a**) and Fe K-edge FT-EXAFS in R space (**b**) for FeN/Fe_3N after CO_2RR at different potentials. Inset (**a**): the enlarge section from 7110 to 7123 eV.

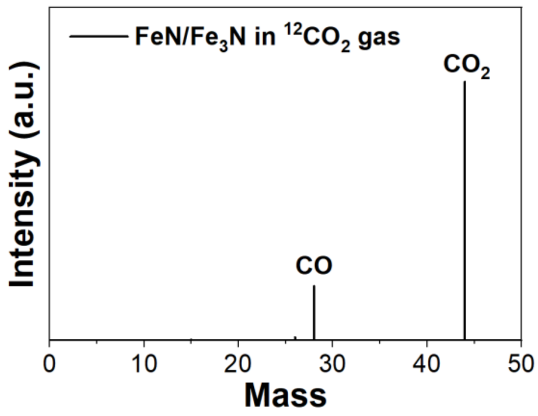

Supplementary Figure 25 | DEMS spectrum of the CO_2RR product catalyzed by FeN/Fe_3N.

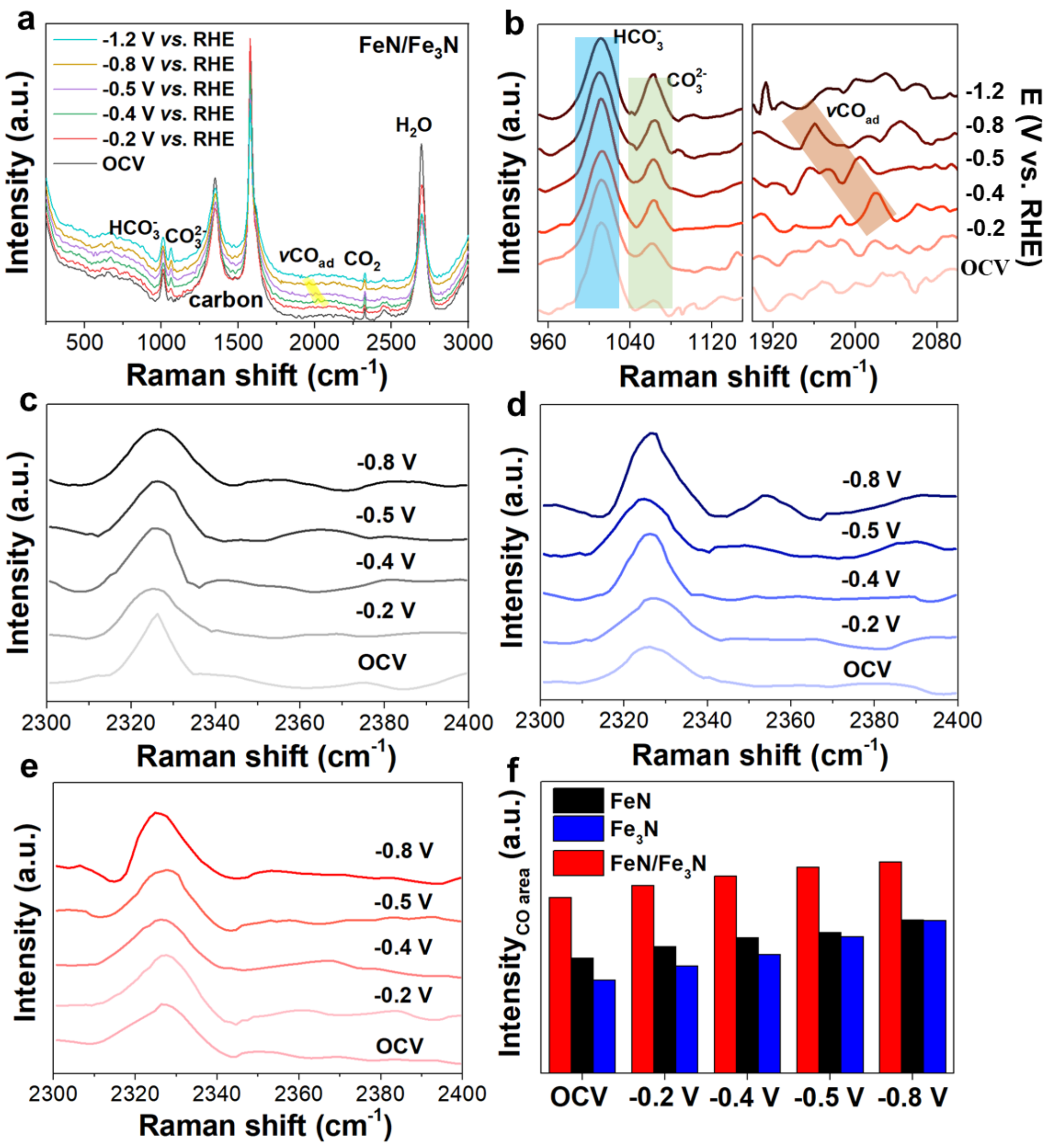

Supplementary Figure 26 | In situ Raman spectra (**a**) and the enlarged peaks of HCO_3^-, CO_3^{2-}, and CO (**b**) present in the FeN/Fe_3N catalyzed CO_2RR. CO_2 peaks for FeN (**c**), Fe_3N (**d**), and FeN/Fe_3N (**c**) of in situ Raman spectra under different potential. **f**, The corresponding CO peak area for FeN-, Fe_3N- and FeN/Fe_3N-catalyzed CO_2RR under different potentials.

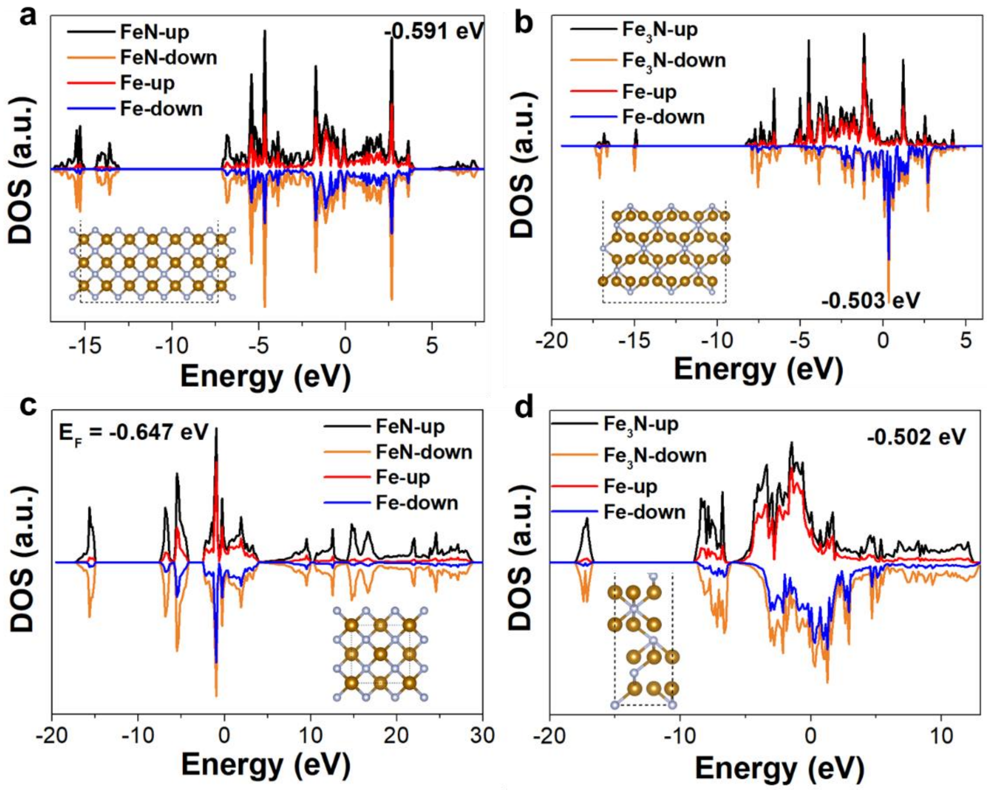

Supplementary Figure 27 | The electronic density of states and d-band centers of Fe in pure FeN (100) (**a**), pure Fe_3N (001) (**b**), FeN cell (**c**), and Fe_3N cell (**d**).

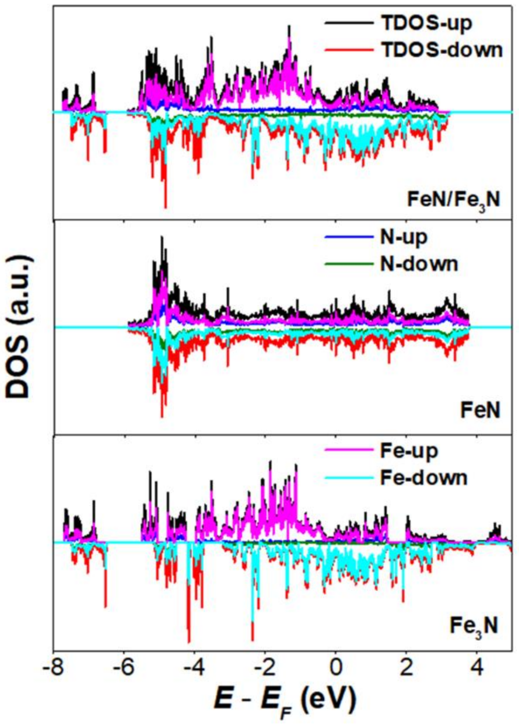

Supplementary Figure 28 | The total and projected electronic density of states of FeN, Fe_3N and FeN/Fe_3N.

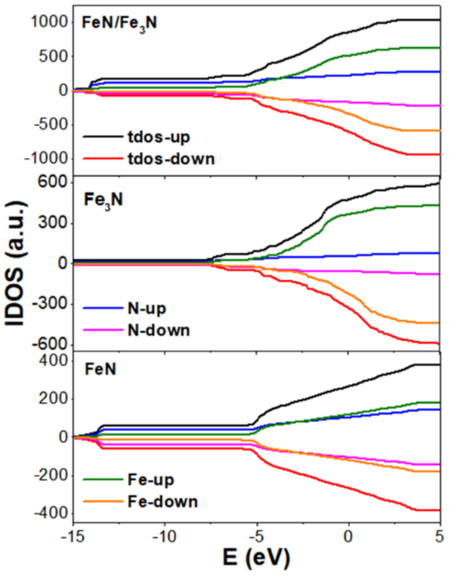

Supplementary Figure 29 | The integral electronic density of states for FeN, Fe_3N and FeN/Fe_3N.

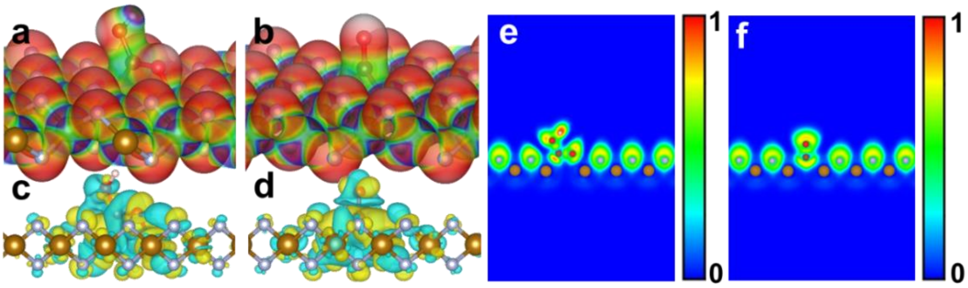

Supplementary Figure 30 | The electrostatic potential for adsorption of COOH (**a**) and CO (**b**) on the FeN surface. 3D electron density difference distributions for adsorption of COOH (**c**) and CO (**d**) on the FeN surface. The electron localization for adsorption of COOH (**e**) and CO (**f**) on the FeN surface.

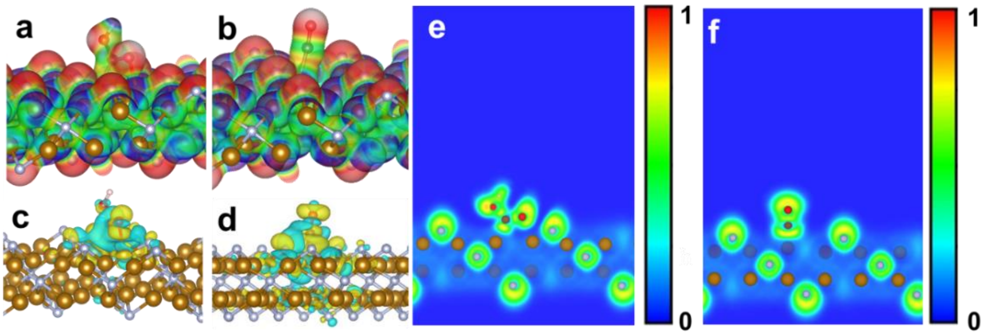

Supplementary Figure 31 | The electrostatic potential for adsorption of COOH (**a**) and CO (**b**) on the Fe_3N surface. 3D electron density difference distributions for adsorption of COOH (**c**) and CO (**d**) on the Fe_3N surface. The electron localization for adsorption of COOH (**e**) and CO (**f**) on the Fe_3N surface.

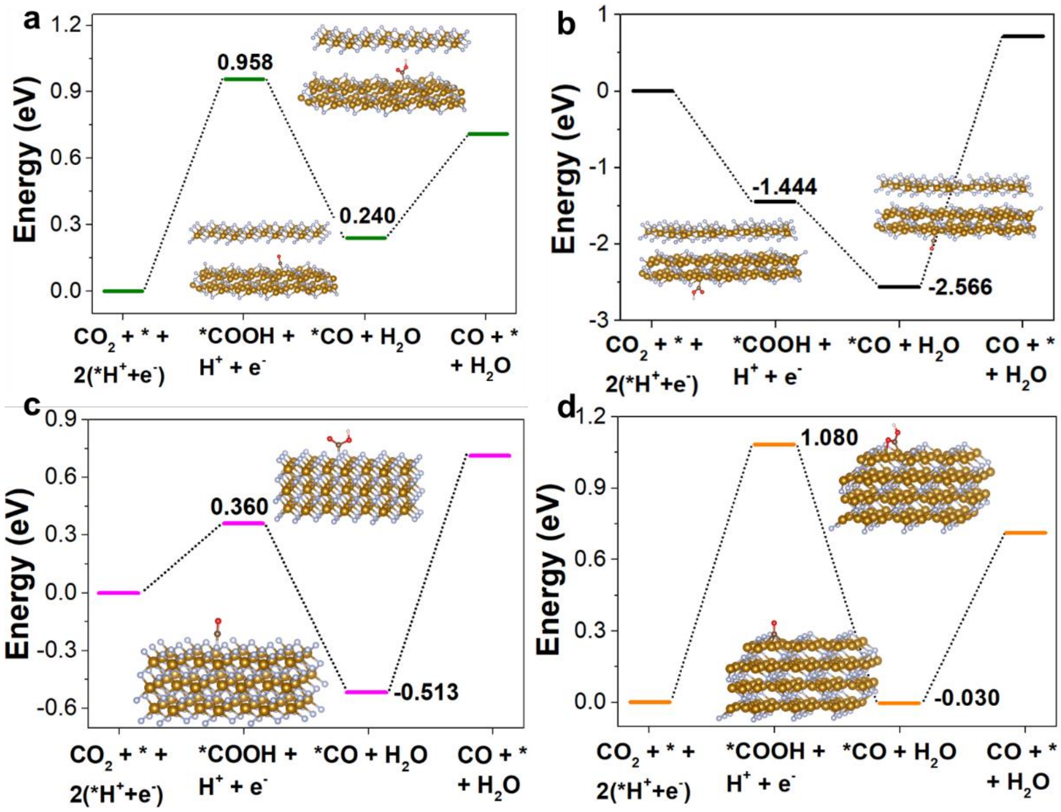

Supplementary Figure 32 | The DFT calculated Gibbs free energy diagrams for CO_2RR on interface Fe_3N (001) (**a**), other side of Fe_3N planes (001) (**b**), pure FeN (100) (**c**), and pure Fe_3N (001) (**d**).

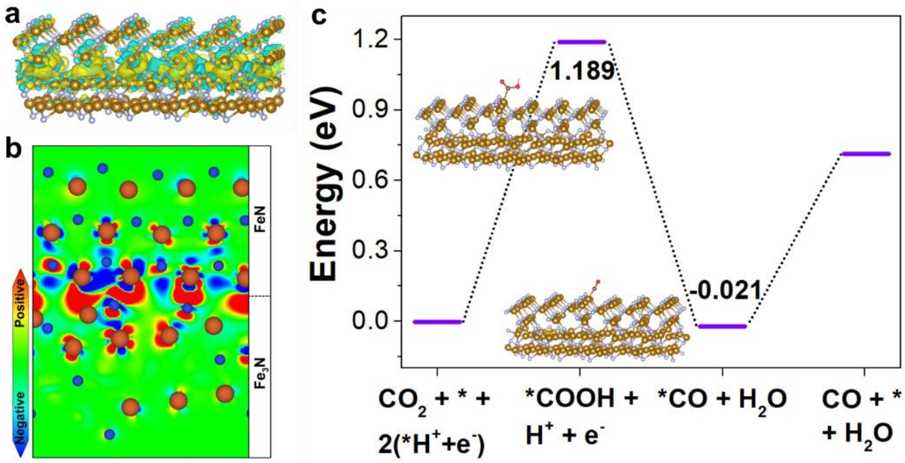

Supplementary Figure 33 | 3D electron density difference distributions (**a**) and the electron localization function of (**b**), the calculated Gibbs free energy diagrams for CO_2RR (**c**) on the FeN(110)/Fe_3N(001) interface.

Supplementary Table 5 | The CO_2 performance of FeN/Fe_3N compared with some representative catalysts.

Catalysts	Electrolyte	FE_{CO}	Potential (V)	j_{CO} (mA cm_{geo}^{-2})	Mass activity (A g^{-1})	TOF (CO s^{-1})	Stability (h)	Ref
FeN/Fe_3N	0.5 M $KHCO_3$	98%	−0.4	21	400 A g_{Fe}^{-1} at −0.4 V	116 CO s^{-1} at −0.9 V	100	This work
Au NWs	0.5 M $KHCO_3$	94%	−0.35	–	1.84 A g_{Au}^{-1} at −0.35	–	12	10
Ni−N−C MOF	0.5 M $KHCO_3$	96.8 %	−0.8	27	–	3.14 CO s^{-1} at −0.8V	10	11
Fe−N_4	0.1M $KHCO_3$	90%	−0.8	25	–	–	–	12
Fe−SAC/ NPC	0.5 M $KHCO_3$	97%	−0.4	5	–	–	24	13
Fe−N_5	0.1M $KHCO_3$	97%	−0.46	1.8	–	–	24	14
Fe_1NC/S_1 −1000	0.5 M $KHCO_3$	96%	−0.5	6.8	–	0.618 CO s^{-1} at −0.65 V	48	15
DNG−SA Fe	0.1M $KHCO_3$	90%	−0.95	33	–	–	19	16
CoPc©Fe-N-C	0.5 M KOH	> 90%	−0.13 to −0.84 V	275.6	–	–	20	17
ZnO and Ag nanoparticles	0.5 M $KHCO_3$	94.1 ± 4.0%	−0.93	22.3	–	259 h^{-1}	150	18
9.4%−Ag alloyed Zn	0.1 M $CsHCO_3$	97%	−1	16.8	–	–	11	19

Supplementary References

1. Guo, W. et al. Atomic Indium Catalysts for Switching CO_2 Electroreduction Products from Formate to CO. *J. Am. Chem. Soc*. **143**, 6877−6885 (2021).

2. Ren, W. et al. Isolated Diatomic Ni-Fe Metal−Nitrogen Sites for Synergistic Electroreduction of CO_2. *Angew. Chem. Int. Ed*. **58**, 6972−6976 (2019).

3. Li, Q. et al. Tuning Sn-Catalysis for Electrochemical Reduction of CO_2 to CO via the Core/Shell Cu/SnO_2 Structure. *J. Am. Chem. Soc*. **139**, 4290−4293 (2017).

4. Rasul, S. et al. A Highly Selective Copper−Indium Bimetallic Electrocatalyst for the Electrochemical Reduction of Aqueous CO_2 to CO†. *Angew. Chem. Int. Ed*. **54**, 2146−2150 (2015).

5. Gu, J. Hsu, C.-S. Bai, L. Chen, H. M. Hu, X. Atomically dispersed Fe^{3+} sites catalyze efficient CO_2 electroreduction to CO. *Science* **364**, 1091−1094 (2019).

6. Guo, Y. et al. Cooperative Stabilization of the [Pyridinium-CO_2-Co] Adduct on a Metal−Organic Layer Enhances Electrocatalytic CO_2 Reduction. *J. Am. Chem. Soc*. **141**, 17875−17883 (2019).
7. Yuan, X. et al. Ultrathin Pd–Au Shells with Controllable Alloying Degree on Pd Nanocubes toward Carbon Dioxide Reduction. *J. Am. Chem. Soc*. **141**, 4791−4794 (2019).
8. Cao, Z. et al. Tuning Gold Nanoparticles with Chelating Ligands for Highly Efficient Electrocatalytic CO_2 Reduction. *Angew. Chem. Int. Ed*. **57**, 12675−12679 (2018).
9. Li, Y. et al. Loading Single-Ni Atoms on Assembled Hollow N-Rich Carbon Plates for Efficient CO_2 Electroreduction. *Adv. Mater*. **34**, 2105204 (2022).
10. Zhu, Z. et al. Active and Selective Conversion of CO_2 to CO on Ultrathin Au Nanowires. *J. Am. Chem. Soc*. **136**, 16132−16135(2014).
11. Jiao, L. et al. Single-Atom Electrocatalysts from Multivariate Metal−Organic Frameworks for Highly Selective Reduction of CO_2 at Low Pressures. *Angew. Chem. Int. Ed*. **59**, 20589−20595 (2020).
12. Adli, N. et al. Engineering Atomically Dispersed FeN_4 Active Sites for CO_2 Electroreduction. *Angew. Chem. Int. Ed*. **60**, 1022−1032 (2021).
13. Sun, X. et al. Phosphorus Induced Electron Localization of Single Iron Sites for Boosted CO_2 Electroreduction Reaction. *Angew. Chem. Int. Ed*. **60**, 23614−23618 (2021).
14. Zhang, H. et al. A Graphene-Supported Single-Atom FeN_5 Catalytic Site for Efficient Electrochemical CO_2 Reduction. *Angew. Chem. Int. Ed*. **58**, 14871−14876(2019).
15. Wang, T. et al. Gas Diffusion Strategy for Inserting Atomic Iron Sites into Graphitized Carbon Supports for Unusually High-Efficient CO_2 Electroreduction and High-Performance Zn−CO_2 Batteries. *Adv. Mater*. **32**, 2002430 (2020).
16. Ni, W. et al. Electroreduction of Carbon Dioxide Driven by the Intrinsic Defects in the Carbon Plane of a Single Fe−N_4 Site. *Adv. Mater*. **33**, 2003238 (2021).
17. Lin, L. et al. Synergistic Catalysis over Iron-Nitrogen Sites Anchored with Cobalt Phthalocyanine for Efficient CO_2 Electroreduction. *Adv. Mater*. **31**, 1903470 (2019).
18. Zhang, Z. et al. "Two Ships in a Bottle" Design for Zn−Ag−O Catalyst Enabling Selective and Long-Lasting CO_2 Electroreduction. *J. Am. Chem. Soc*. **143**, 6855−6864 (2021).
19. Lamaison, S. et al. High-Current-Density CO_2-to-CO Electroreduction on Ag-Alloyed Zn Dendrites at Elevated Pressure. *Joule* **4**, 395−406 (2020).
